# Effect of Fullerenol C_60_(OH)_24_ on the Viability and Metabolism of THP-1 Cells

**DOI:** 10.3390/molecules30224407

**Published:** 2025-11-14

**Authors:** Darya Usanina, Svetlana Zamorina, Maria Bochkova, Valeria Timganova, Violetta Vlasova, Valeria Ponomareva, Maria Dolgikh, Sergey Lazarev, Mikhail Rayev

**Affiliations:** 1 Perm Federal Research Center of the Ural Branch of the Russian Academy of Sciences, Institute of Ecology and Genetics of Microorganisms, 614081 Perm, Russiamantissa7@mail.ru (S.Z.); dolgikh.md@yandex.ru (M.D.); lasest1999@gmail.com (S.L.); 2 Department of Biology, Perm State University, 614086 Perm, Russia

**Keywords:** fullerenol, nanoparticles, THP-1, monocyte, cytotoxicity, apoptosis, metabolism, glycolysis, oxidative phosphrylation

## Abstract

Fullerenols are polyhydroxylated derivatives of fullerene (C_60_(OH)_n_) with antioxidant, antiviral, and antibacterial properties and potential biomedical applications due to their solubility and biocompatibility. However, comprehensive assessment of their cytotoxicity is required, particularly regarding their effects on immune system cells. This study investigated the effects of fullerenol C_60_(OH)_24_ (MST-Nano, St. Petersburg, Russia) on the viability, apoptosis, and metabolism of THP-1 human monocytic leukemia cells. Cells were treated with concentrations ranging from 0.25 to 1000 µg/mL and incubated for 24, 48, and 72 h. Viability, apoptosis, and nanoparticle association were assessed by flow cytometry; glycolysis and mitochondrial respiration were measured after 24 h on a Seahorse XFe96 analyzer (Agilent Technologies, Santa Clara, CA, USA). Results showed that the effects of fullerenol depend on concentration and exposure time. At 24 h, 750 µg/mL increased viability, while 1000 µg/mL induced apoptosis. After 48 and 72 h, apoptosis increased at concentrations ≥750 µg/mL, with reduced viability. Nanoparticle association correlated with concentration and inversely correlated with viability but was independent of incubation time. Metabolic analysis revealed decreased glycolysis at 750 µg/mL after 24 h, while mitochondrial respiration was unaffected. Thus, our study demonstrated that fullerenol nanoparticles were safe for the THP-1 monocytic cell line up to 500 µg/mL.

## 1. Introduction

One of the promising and rapidly developing areas of modern biomedicine is the study of nanomaterials as therapeutic and diagnostic agents. Due to their great diversity and ease of modification, nanomaterials can be adapted for a wide range of applications. Carbon-based nanomaterials, including graphene, nanodiamonds, carbon quantum dots, carbon nanotubes, fullerenes, and others, attract significant interest. The most common form of fullerene, C_60_, consists of carbon atoms arranged in a spherical structure. This material is attractive for biomedical research, particularly because of its significant antioxidant properties; however, its low solubility limits its applications [1]. Functionalization of fullerene C_60_ with hydroxyl groups produces fullerenols, C_60_(OH)_n_ (2 ≤ *n* ≤ 44), which are water-soluble and more suitable for biological use [2]. Fullerenols have potential applications in agriculture and as antioxidants, therapeutic agents (due to their antiviral and antibacterial activities), photosensitizers, drug carriers, and biosensors [2,3]. Currently, several fullerene-based products are registered in cosmetology and ophthalmology [4].

The introduction of nanoparticle-based drugs into biomedicine necessitates a comprehensive assessment of their toxicity. One key factor influencing the cytotoxicity of fullerenols is the degree of hydroxylation. For instance, Kovel et al. [5] reported that fullerenols with 24–28 hydroxyl groups exhibit lower toxicity and higher antioxidant activity compared to those with fewer hydroxyl groups. Furthermore, the same research group [6] suggested that the reduced toxicity and enhanced antioxidant properties of the fullerenols C_60_(OH)_24–28_ may be attributed to their increased solubility.

Non-functionalized fullerenes are known to be cytotoxic. For example, pure fullerene C_60_ induces rapid necrosis in tumor cell lines L929 (mouse), C6 (rat), and U251 (human) via the generation of reactive oxygen species [7]. In contrast, hydroxylated fullerene C_60_(OH)_n_ exerts only a moderate pro-apoptotic effect that is not associated with reactive oxygen species. Subsequent studies confirm that fullerenol C_60_(OH)_24_ exhibits low genotoxicity and cytotoxicity, indicating high biocompatibility [8]. This enhanced biocompatibility is attributed to the increased water solubility and reduced oxidative stress potential of hydroxylated derivatives, making fullerenols promising candidates for biomedical applications. However, toxicity can depend on factors such as degree of hydroxylation and concentration, with higher hydroxylation generally correlating with lower toxicity but potentially increased cellular interactions.

A crucial aspect of evaluating the cytotoxicity of any material is assessing its immunocompatibility, as immune cells actively respond to foreign substances introduced into the body. Although studies on fullerenols remain limited, they have demonstrated notable immunomodulatory effects [9], high biocompatibility [10], and the ability to enhance phagocytosis [11,12].

At the same time, given the pronounced antioxidant properties of fullerenol, its stability, and its ability to exert antiviral and antitumor effects, we see its high potential in biomedicine [13].

However, these investigations have employed fullerenol variants with differing degrees of hydroxylation, which complicates data interpretation and hinders a comprehensive understanding of the cytotoxic effects of specific fullerenol particles on distinct immune cell subpopulations.

Our study aims to address this gap by systematically investigating the effects of fullerenol nanoparticles C_60_(OH)_24_ on various subpopulations of human immune cells. This research focuses on the THP-1 cell line, a model of human acute monocytic leukemia. In the initial phase, we sought to determine the impact of fullerenol nanoparticles C_60_(OH)_24_ at varying concentrations and incubation times on the viability, apoptosis, and metabolism of monocytic cells, as well as to evaluate nanoparticle uptake by these cells. Identifying cytotoxic concentration thresholds in this study will facilitate more targeted and effective future investigations into the effects of fullerenol on additional functional parameters of monocytic cells and other immune cell subpopulations.

## 2. Results

### 2.1. Characterization of Nanoparticles

**Figure 1:**
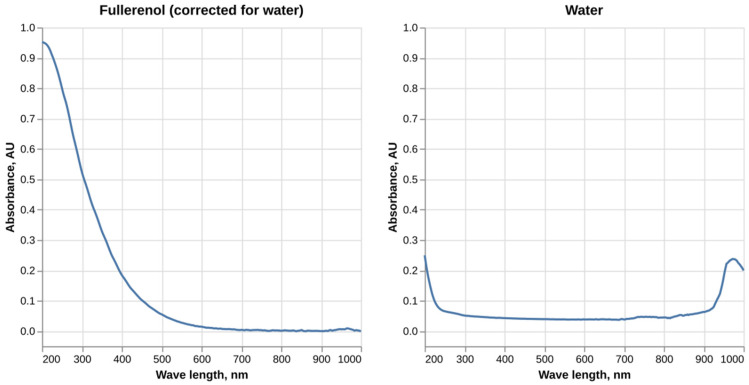
UV-Vis absorbance spectrum of fullerenol C_60_(OH)_24_ solution in water.

Our data on the absorbance spectrum of fullerenol C_60_(OH)_24_ in the UV-Vis range are presented in Figure 1. Fullerenol solution absorbs light in the broad spectrum of 200–600 nm with the absorbance value monotonously decreasing. At the same time no absorbance in the red part of the spectrum was observed.

**Figure 2:**
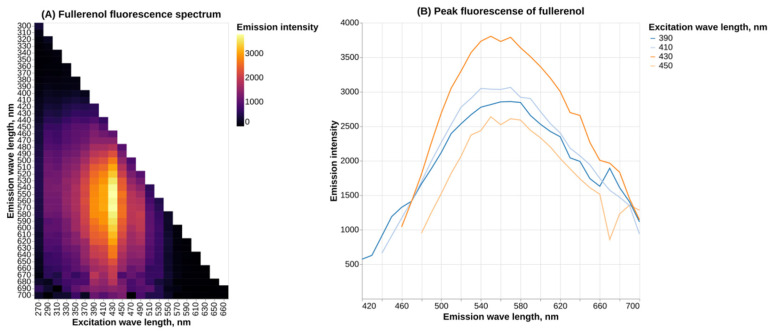
Fluorescence spectrum of fullerenol C_60_(OH)_24_ in water.

Fullerenol C_60_(OH)_24_ demonstrates a broad fluorescence spectrum, with the highest intensity of fluorescence occurring at the λ_ex_ = 430 nm and an emission maximum at around 560 nm (Figure 2).

**Figure 3:**
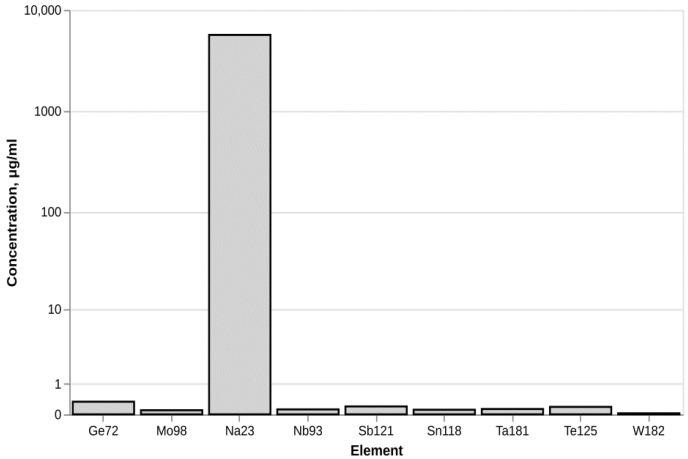
Metal impurities in fullerenol C_60_(OH)_24_.

Analysis of metal impurities using ICM-MS determined the presence of sodium in the fullerenol sample (Figure 3). At the same time the concentrations of other metal elements remained at the background level.

A linear calibration curve for the determination of endotoxin content was obtained with the R^2^ value of 0.982. Fullerenol concentration in the sample containing 10 μg/mL of nanoparticles was measured to be 0.012 EU/mL, which corresponds to the endotoxin content of 1.2 × 10^−3^ EU/μg for dry fullerenol.

**Figure 4:**
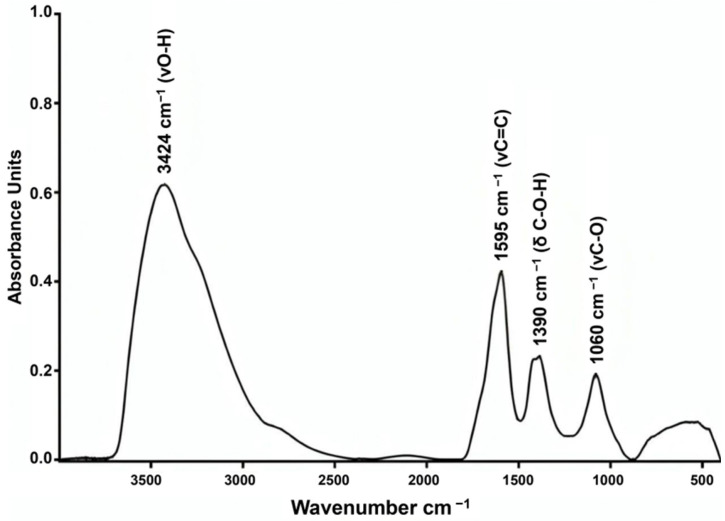
IR spectrum of fullerenol C_60_(OH)_24_.

The IR spectrum exhibits the following characteristic absorption bands (Figure 4): 3424 cm^−1^ (O–H stretching vibrations), 1595 cm^−1^ (C=C bending vibrations), 1390 cm^−1^ (bending vibrations of the C–O–H functional group), and 1060 cm^−1^ (stretching vibrations of the C–O functional group).

**Figure 5:**
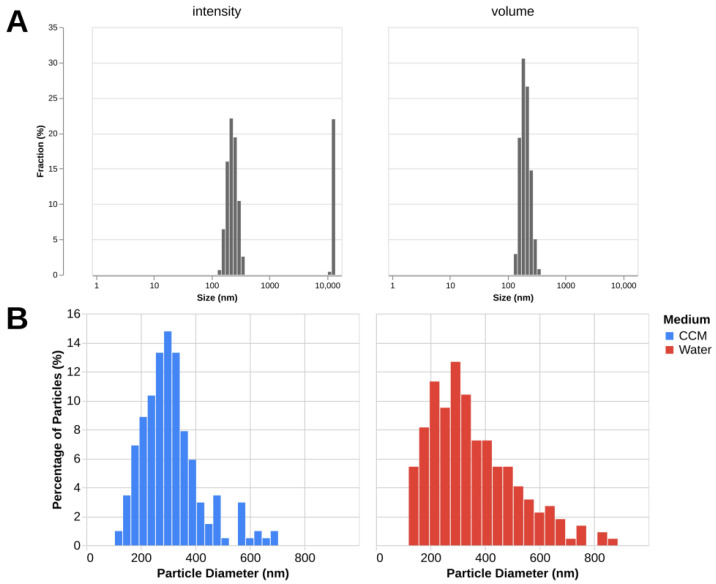
C_60_(OH)_24_ determined by DLS and TEM. (**A**)—Intensity- and volume-based distributions as measured by DLS. Mean values for 3 measurements are displayed. (**B**)—Particle diameter distributions measured by TEM in water (222 measurements) and complete culture medium (203 measurements).

Size measurement of fullerenol nanoparticles using DLS in water shows that the volume-based particle diameter is 186 nm with the polydispersity index (PDI) of 0.45. It can be noted from the plot (Figure 5) that intensity-based size distribution shows the presence of large aggregates absent in the volume-based distribution, which can be attributed to the fact that intensity-based metrics are more sensitive to the presence of large particles. The zeta-potential of a 100 μg/mL solution of fullerenol in distilled water is −26 ± 6.31 mV (mean ± SD).

The mean particle diameters obtained from TEM images are 356 ± 167 (mean ± SD) in water and 308 ± 110 (mean ± SD) in the complete culture medium (Figure 5). The data show a wide distribution of sizes, which is consistent with DLS. The discrepancy between the mean diameter values of the DLS and TEM measurements is expected as the methods measure different parameters (hydrodynamic diameter and core diameter) of the samples in different states (dissolved in water and dried).

### 2.2. Effect of Fullerenol C_60_(OH)_24_ on the Viability and Apoptosis of THP-1 Cells

**Figure 6:**
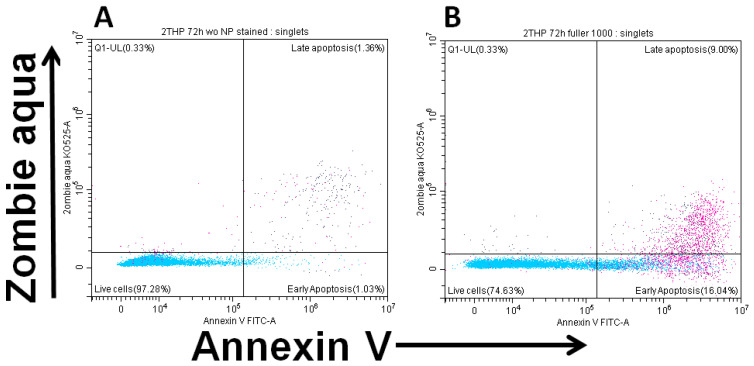
Early (ZA^−^Ann^+^) and late (ZA^+^Ann^+^) apoptosis of THP-1 cell line cells. (**A**)—culture without fullerenol addition, (**B**)—culture with 1000 µg/mL fullerenol added.

Figure 6 demonstrates the changes in viability and apoptosis in cultures upon the addition of the maximum concentration of fullerenol.

**Figure 7:**
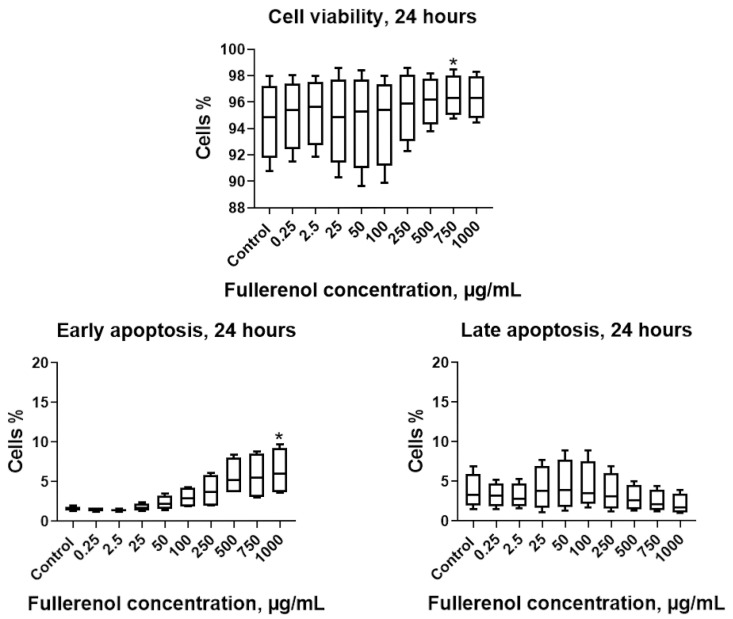
Effect of fullerenol nanoparticles on the viability and apoptosis (early, late) of THP-1 cell line cells after 24 h of incubation. Control—cultures without nanoparticles. Significant differences (*, *p* < 0.05) compared to the control are indicated according to the Friedman test and Dunn’s post hoc test for multiple comparisons. Data are presented as median, lower and upper quartiles, minimum, and maximum. *n* = 4.

It was found that after 24 h of incubation, fullerenol concentrations up to 750 µg/mL did not adversely affect the parameters studied. Notably, at 750 µg/mL, fullerenol significantly increased cell viability (Q1–Q3: 95.0–98.0% vs. 91.8–97.2% in the control). However, increasing the fullerenol concentration to 1 mg/mL resulted in a significant rise in the proportion of cells undergoing early apoptosis (Figure 7).

**Figure 8:**
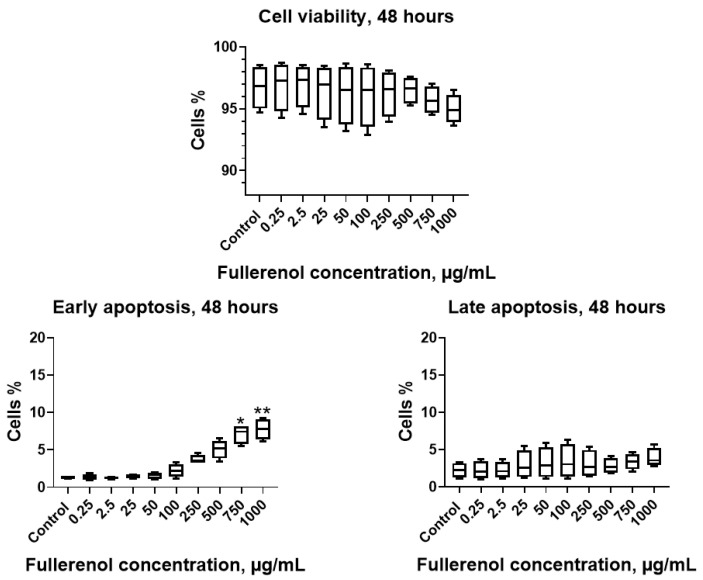
Effect of fullerenol nanoparticles on the viability and apoptosis (early, late) of THP-1 cell line after 48 h of incubation. Control—cultures without nanoparticles. Significant differences (*, *p* < 0.05; **, *p* < 0.01) compared to the control are indicated according to the Friedman test and Dunn’s post hoc test for multiple comparisons. Data are presented as median, lower and upper quartiles, minimum, and maximum. *n* = 4.

When the incubation period was extended to 48 h, no significant effect of the nanoparticles on cell viability was observed; however, concentrations of 750–1000 µg/mL induced a significant increase in the proportion of cells undergoing early apoptosis (Figure 8).

**Figure 9:**
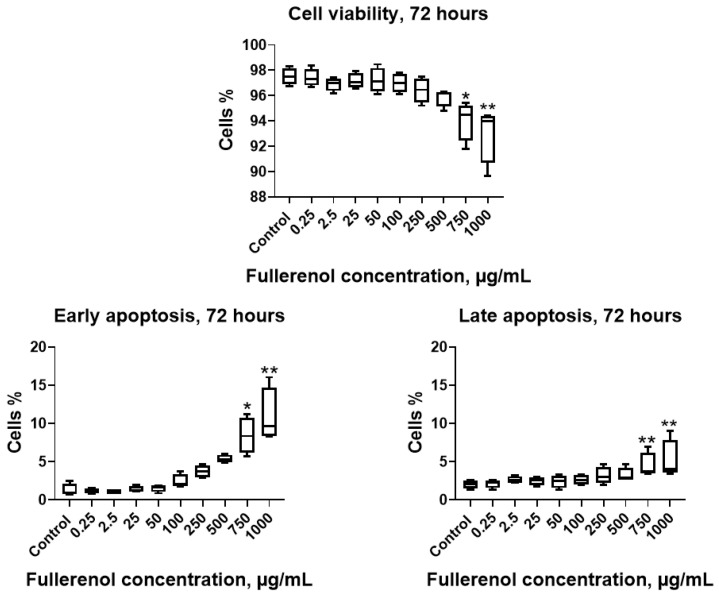
Effect of fullerenol nanoparticles on the viability and apoptosis (early, late) of THP-1 cell line after 72 h of incubation. Control—cultures without nanoparticles. Significant differences (*, *p* < 0.05; **, *p* < 0.01) compared to the control are indicated according to the Friedman test and Dunn’s post hoc test for multiple comparisons. Data are presented as median, lower and upper quartiles, minimum, and maximum. *n* = 4.

Co-culturing the THP-1 cell line with fullerenol for 72 h at concentrations of 750–1000 µg/mL resulted in a significant decrease in cell viability accompanied by increased apoptosis, including both early and late stages (Figure 9).

### 2.3. Cell Association of Fullerenol C_60_(OH)_24_

**Figure 10:**
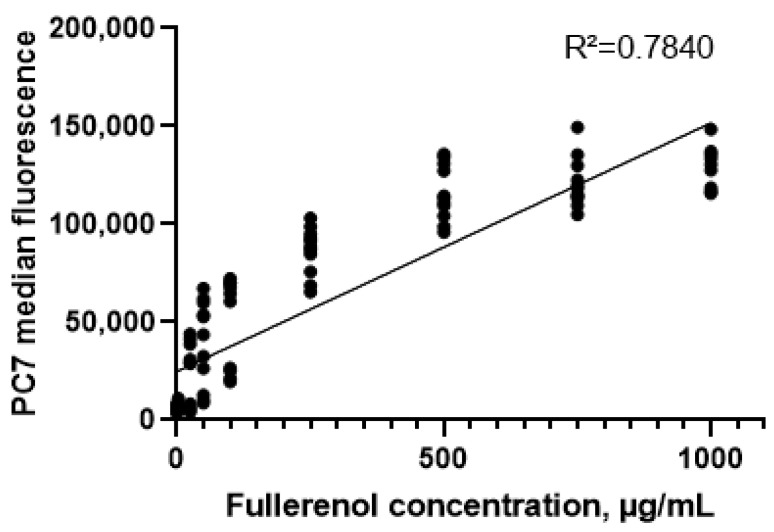
Correlation between the concentration of fullerenol nanoparticles in cultures and the median fluorescence in the PC7 channel (24–72 h).

It was established that fullerenol is taken up or adsorbed by the cells, as indicated by an increase in the median fluorescence intensity in the PC7 (Pe-Cy7, phycoerythrin-cyanine 7) channel with rising fullerenol concentrations. A strong positive correlation was observed between median fluorescence intensity and fullerenol concentration in the cultures (Spearman’s correlation coefficient, r = 0.94 (95% confidence interval: 0.91–0.96), *p* < 0.0001) (Figure 10).

**Figure 11:**
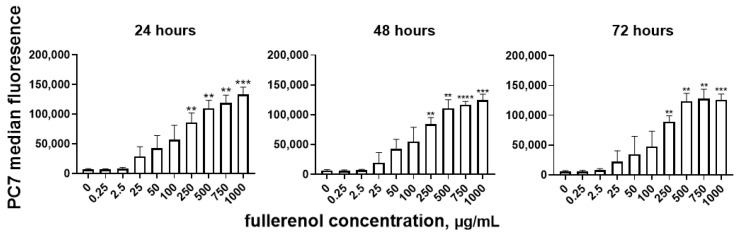
Cell association of fullerenol C_60_(OH)_24_ at different cultivation times. Significant differences are indicated (**, *p* < 0.01; ***, *p* < 0.001; ****, *p* < 0.0001) compared to the control (0) according to Welch’s/Brown–Forsythe test. *n* = 4. M ± SD.

A statistically significant increase in median fluorescence intensity in the PC7 channel was observed when the fullerene concentration reached 250 μg/mL (Figure 11). Analysis by two-way ANOVA revealed that only nanoparticle concentration significantly affected the level of fullerenol association (*p* < 0.0001), whereas the duration of co-cultivation had no significant effect (*p* = 0.37).

Next, correlation analysis was performed to examine the relationships between fullerenol association and cell viability or apoptosis at different incubation times (Table 1). After 24 h of incubation, no significant correlation was observed between median PC7 fluorescence intensity and cell viability. However, a weak negative correlation emerged at 48 h, which strengthened to a strong negative correlation by 72 h. In contrast, a statistically significant positive correlation between fullerenol association and cell apoptosis was detected at all incubation times, with the correlation strength increasing over time.

### 2.4. Effect of Fullerenol C_60_(OH)_24_ on the Metabolism of THP-1 Cells

After 24 h, concentrations ranging from 2.5 to 1000 µg/mL did not exhibit cytotoxic effects on the cells; however, the 1000 µg/mL concentration did show pro-apoptotic activity. We aimed to investigate the effects of non-apoptotic concentrations of fullerenol (2.5–750 µg/mL) on the metabolism of THP-1 cells to identify any earlier metabolic effects, if present.

**Figure 12:**
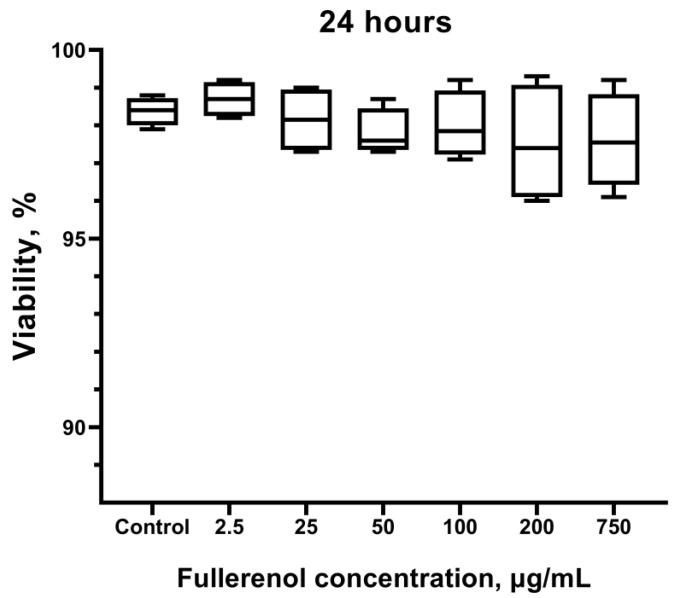
Effect of fullerenol nanoparticles on the viability of THP-1 cells. Cells not treated with fullerenol are used as control. Data are presented as median, lower, and upper quartiles, minimum, and maximum. *n* = 4.

No statistically significant differences in cell viability were observed compared to the control group (not treated with the nanoparticles) after culturing with fullerenol (Figure 12). This finding is consistent with the data obtained by flow cytometry (Figure 7), except at the fullerenol concentration of 750 µg/mL, which exhibited a stimulating effect as indicated by Zombie Aqua staining.

**Figure 13:**
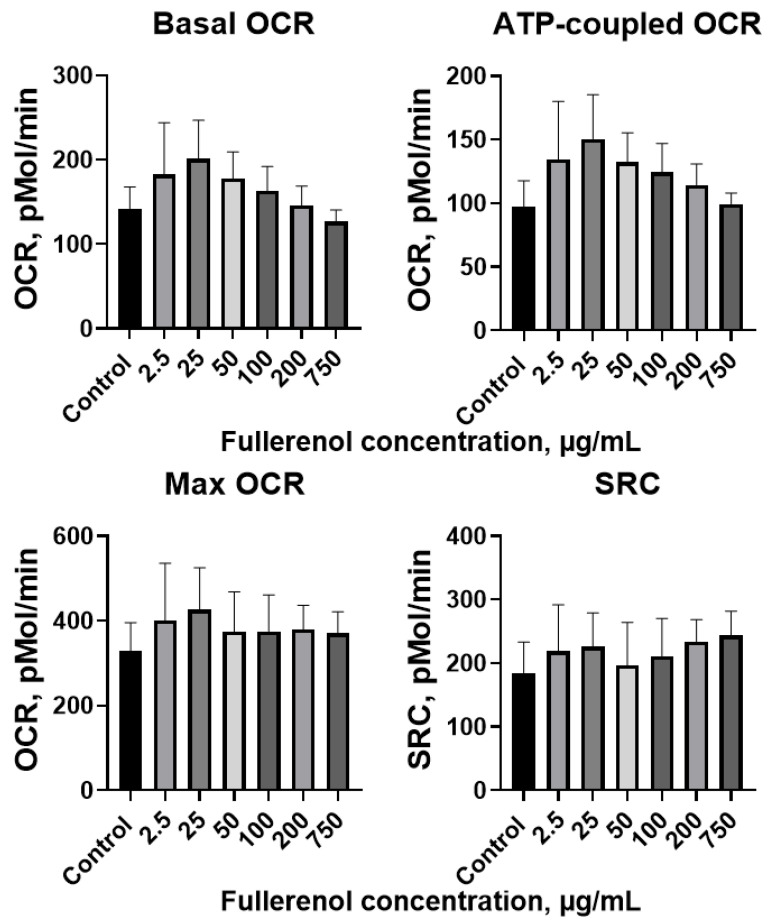
Basal, ATP-coupled, and maximal oxygen consumption rates (OCR), and spare respiratory capacity (SRC) of THP-1 cells co-cultured with fullerenol nanoparticles for 24 h. M ± SD. *n* = 4.

Metabolic analysis revealed that 24 h incubation with fullerenol did not significantly impact mitochondrial respiration in THP-1 cells. Basal OCR (oxygen consumption rates), ATP-coupled respiration, max OCR, and SRC (spare respiratory capacity) remained comparable to control values across all tested fullerenol concentrations (Figure 13).

**Figure 14:**
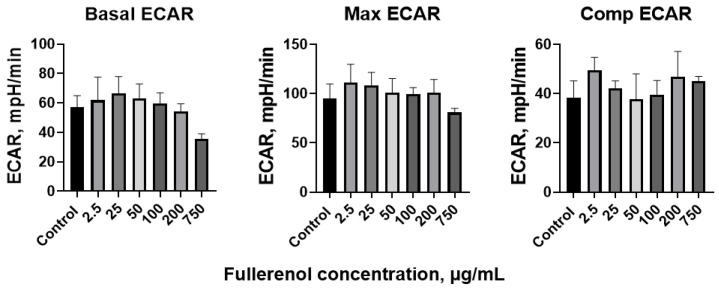
Basal, maximal, and compensatory extracellular acidification rate (ECAR) of THP-1 cells co-cultured with fullerenol nanoparticles for 24 h. M ± SD. *n* = 4.

After 24 h of incubation with fullerenol, the ECAR values in the THP-1 cells showed no significant changes. Basal, maximal, and compensatory ECAR levels were similar across all tested fullerenol concentrations (Figure 14). 

**Figure 15:**
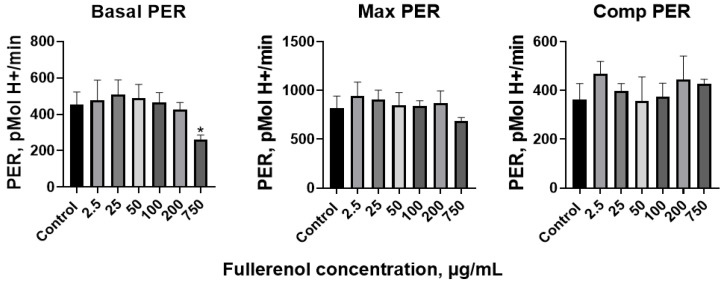
Basal, maximal, and compensatory proton efflux rate (PER) of THP-1 cells co-cultured with fullerenol nanoparticles for 24 h. M ± SD. *n* = 4. *p* values < 0.05 are indicated (*).

However, the proton efflux rate (PER) decreased at the highest concentration of 750 µg/mL (Figure 15). The maximal and compensatory PER remained stable across all concentrations tested.

Thus, fullerenol has virtually no modulatory effect on metabolism in THP-1 cells, with the exception of Basal PER, which was decreased, and the glycolytic rate, which was reduced after 24 h of incubation with fullerenol at a concentration of 750 µg/mL.

## 3. Discussion

The absorbance spectrum of fullerenol obtained in this study is consistent with the data published by other authors. Similar results were obtained by Pritam et al. [14] (see Supplementary Materials); however, in their study fullerenol showed detectable absorbance only up to 350 nm, most likely due to a lower concentration of nanoparticles being used. Another two studies show a slightly different shape of the absorbance spectrum with the absorbance value increasing in the 200 nm region before starting to monotonously decrease [15,16]. A study by Vileno et al. suggests that the absorption spectrum of fullerenol might be concentration-dependent, with some concentrations displaying monotonous absorbance change while others having more complex patterns [17].

Our results on the fullerenol fluorescence spectrum also generally agree with existing data. In a previous study, peak emission of fullerenol at λ_ex_ = 420 nm was shown to be at 470 nm, which does not match present results [18]. We speculate that the observed discrepancy might be due to differences in the experimental setup (pH and ionic strength of the solvent and fullerenol concentration). Another study demonstrates that the most intense emission occurs with a wavelength of around 525 nm, which is closer to our data [19], although it should be noted that this study used the excitation wavelength of 340 nm. Another article shows peak fullerenol fluorescence at about 550 nm with λ_ex_ = 300 nm [15] (see Supplementary Materials). All of the cited studies only examined the fullerenol emission spectrum at a single excitation wavelength, while the present work provides a more comprehensive analysis.

In regard to the metal impurities, it is known that sodium ions can be present in fullerenol nanoparticles when certain synthesis methods are used for their preparation [17]. No evidence has been presented in the literature that the presence of sodium affects the biological properties of the nanoparticles.

LAL test results showed that the fullerenol endotoxin content is 1.2 × 10^−3^ EU/μg. Considering that in our in vitro experiments nanoparticle concentrations of 0.25 to 200 μg/mL were used, the maximal endotoxin level to which the cells were exposed would have been 0.24 EU/mL. This value is lower than the recommended threshold (<0.25 EU/mL) used in the manufacture of Water for Injection [20]. The FBS used in this study contained not more than 5 EU/mL of endotoxin according to the certificate of analysis provided by the manufacturer, which given its final concentration of 10% would mean that the addition of serum could introduce up to 0.5 EU/mL of endotoxin in the cell culture medium—a level comparable to that of fullerenol. It is worth noting that the net endotoxin level introduced by the use of serum (up to 0.5 EU/mL) may have been above pharmacopeial limits, but it does not invalidate the results as the control also contained an equivalent amount of serum. Moreover, the use of FBS in immunological research is a standard practice which is used widely among many laboratories.

The features of the obtained IR spectrum are consistent with polyhydroxylated fullerenes and correlate well with data presented in the literature [21,22]. The obtained IR is indicative of high purity of the fullerenol used in this study and matches the spectrum provided by the manufacturer.

The data on the size of fullerenol presented in the literature show a wide range of possible values [13], and our results fall within this range. The average PDI is 0.45, which indicates that the particles exhibit a high degree of polydispersity with the generally recommended value for clinical application being <0.3 [23]. Polydispersity is especially evident from the intensity-based size distribution, which shows the presence of large aggregates in the sample despite extensive ultrasound treatment.

Overall, despite the fact that fullerenol characterization methods are not standardized between different studies, we can conclude that the nanoparticles used herein possess physico-chemical characteristics which are generally similar to those reported in the literature. In addition, we have identified that our fullerenol contains sodium impurities. It also exhibits a low endotoxin level, which makes it suitable for tests involving immune cells.

The results indicate that the effect of fullerenol on THP-1 cells depends on both particle concentration and incubation time. Nanoparticles at concentrations up to and including 500 μg/mL did not exhibit cytotoxic effects regardless of the incubation duration. At 750 μg/mL, a statistically significant increase in cell viability was observed after 24 h; however, after 48 to 72 h, the same concentration induced cytotoxic effects.

In a previous study, we investigated the cytotoxicity of the same fullerenol C_60_(OH)_24_ nanoparticles on human peripheral blood natural killer (NK) cells using a similar approach [24]. Nanoparticles at concentrations ranging from 0.2 to 200 μg/mL exhibited no toxicity to NK cells regardless of the incubation period (24–72 h), consistent with the findings of the current study. Additionally, it was demonstrated that fullerenol was associated with NK cells at higher concentrations (100 and 200 μg/mL), with the proportion of fullerenol (PC7)-positive cells increasing with both longer incubation times and higher nanoparticle concentrations. Interestingly, a similar increase in Fullerenol association with rising particle concentration was observed in the THP-1 cell line; however, statistically significant effects occurred only at higher doses (≥250 μg/mL), and no dependence of fluorescence on incubation time was detected. Moreover, we found that fullerenol C_60_(OH)_24_ at concentrations of 100 and 200 µg/mL was associated with T and B lymphocytes, exerting cytotoxic effects only on T cells and only at the concentration of 200 µg/mL [25]. For normal peripheral blood monocytes, we determined a minimum cytotoxic concentration of 200 µg/mL following incubation periods of 24 to 72 h. The viability of monocytes decreased by approximately 20–30%, showing an inverse correlation with the proportion of cell-associated fullerenol [26]. The comparative summary of the effects of fullerenol C_60_(OH)_24_ on various subpopulations of immune cells in our studies is presented in Table 2.

Comparing our data with the existing literature presents several challenges. Different research groups often use fullerenol nanoparticles with varying degrees of hydroxylation, and the precise chemical formulas of the particles are not always specified. Additionally, particle concentrations vary widely across studies, and incubation times rarely exceed 48 h. Despite these limitations, Ravelo-Nieto et al. [27] demonstrated that fullerenol nanoparticles do not exert toxic effects on THP-1 cells after 24 and 48 h co-cultivation at concentrations ranging from 18.7 to 300 μg/mL, which aligns with the findings of our study.

Fullerenol C_60_(OH)_36_ at concentrations of 50–150 μg/mL did not affect the viability or apoptosis of mononuclear cells after 24 h of incubation [28], nor did it impact dog kidney cells at concentrations up to 200 μg/mL over the same period [29]. Studies on various other cell lines—including retinal epithelium, retinal glial cells, umbilical vein endothelial cells, keratinocytes, fibroblasts, osteoblast precursors, cardiomyocytes, and mouse epithelium—as well as human peripheral blood mononuclear cells and rat tendon cells, similarly reported no toxic effects of fullerenol [2]. Particle concentrations in these studies ranged broadly from 0.1 to 1000 μg/mL.

In contrast, Isakovic et al. reported an LC50 of 800–1000 μg/mL for fullerenol after 24 h incubation in mouse fibrosarcoma, rat glioma, and human glioma cell lines [7]. Notably, fullerenol enhanced caspase-dependent apoptosis characterized by DNA fragmentation and loss of membrane asymmetry but did not induce necrosis. However, the authors did not specify the degree of hydroxylation of the fullerenol particles used, which complicates direct comparison with other studies.

At low doses, fullerenol has been shown to enhance cell characteristics, as observed on the first day of incubation in our study and similarly reported by Zha et al. in hippocampal neurons [30].

Nanoparticle uptake by cells is known to be associated with various cytotoxic effects, including oxidative stress, apoptosis, autophagy, and inflammation [31]. Fullerenol cannot passively diffuse across cellular membranes [32]; however, fullerenol C_60_(OH)_~30_ has been shown to adhere to the membranes of erythrocytes [33]. Moreover, fullerenol is capable of penetrating the cytoplasm and nucleus [34], the perinuclear cytoplasm [35], endosomes [36], and lysosomes [37]. Uptake of fullerenol increases with rising particle concentration [28], consistent with our findings. Notably, in that study—as well as in our previous research on NK cells—particle uptake by mononuclear cells also increased with longer incubation times.

While our study does not distinguish between adhesion and internalization of fullerenol, existing literature suggests that prolonged incubation alters the ratio of adhered versus internalized particles, which may contribute to decreased cell viability and increased apoptosis. Previous studies have reported the ability of fullerenol to inhibit tumor growth and metastasis [35,38,39].

One of the key findings of this study was the effect of high concentrations of fullerenol on the metabolism of THP-1 cells. After 24 h of incubation with 750 µg/mL fullerenol, glycolysis in THP-1 cells was significantly decreased. This observation is important because glycolysis serves as the primary metabolic pathway for many cancer cells, providing rapid energy and biosynthetic intermediates essential for their growth and survival. However, the precise mechanism through which fullerenol influences cellular metabolism remains to be elucidated.

Previously, using colocalization analysis of endosomes/lysosomes and nanoparticles, it was shown that the fullerenol conjugate with buforin-II (Buf II) is capable of escaping from the endosomes of THP-1 cells into the cytosol. The authors suggest that the most likely mechanisms for nanoparticle escape from endosomes are the formation of transient pores or the proton sponge effect [27]. It can be assumed that once in the cytosol, the nanoparticles may interact with enzymes involved in glycolysis, causing their inactivation. There is evidence of the ability of fullerenol and other fullerene derivatives to inhibit the activity of enzymes that are highly expressed in cancer cells, such as glutathione-S-transferase, tyrosine protein phosphatases, tyrosine kinases, caspases, and metalloproteinases [40,41]. The physico-chemical characteristics of C_60_ fullerene-based nanomaterials allow them to adsorb onto the protein surface, penetrate into hydrophobic pockets of proteins, and alter their conformation [42].

## 4. Materials and Methods

### 4.1. Cell Line

The object of this study was the THP-1 cell line (acute monocytic leukemia) obtained from “PrimeBioMed,” (Moscow, Russia). THP-1 cells were stored in liquid nitrogen vapor at −196 °C. Prior to the experiment, the cells were thawed in a water bath, washed to remove the cryopreservation medium (fetal bovine serum containing 10% dimethyl sulfoxide), and cultured in complete culture medium (CCM) to reach the exponential growth phase. The CCM consisted of RPMI-1640 (Gibco, Waltham, MA, USA) supplemented with 2 mM L-glutamine (Capricorn, Ebsdorfergrund, Germany), 100 units/mL penicillin, 0.1 mg/mL streptomycin, 2.5 µg/mL amphotericin B (BI, Kibbutz Beit Haemek, Israel), and 10% heat-inactivated fetal bovine serum (Capricorn, Ebsdorfergrund, Germany). Cell viability was monitored throughout the culturing process.

### 4.2. Fullerenol C_60_(OH)_24_

The study utilized fullerenol C_60_(OH)_22–24_ (MST-WS60-Bio, Modern Synthesis Technology, St. Petersburg, Russia). Since the content of C_60_(OH)_22_ declared by the manufacturer in the fullerenol C_60_(OH)_22–24_ preparation was ≈ 0.01%, the fullerenol is designated only by the formula C_60_(OH)_24_.

### 4.3. Z-Potential and DLS Size Measurement

Fullerenol was dissolved in distilled water and diluted to the final concentration of 200 μg/mL. The sample was treated with ultrasound for 30 min at a 70% power setting. Nanoparticle size was measured using dynamic light scattering (DLS) with BeNano 180 Zeta Pro (Bettersize, Dandong, China) in a polystyrene cuvette at room temperature. The measurement angle was set to 90°. Volume-based particle size distributions were obtained and the median values of three measurements were calculated. The size value exhibiting the highest frequency is reported. The zeta-potential of a 100 μg/mL solution of fullerenol in distilled water was measured using BeNano 180 Zeta Pro (China) in a polystyrene cuvette at room temperature. Six measurements were performed; mean value and standard deviation are reported.

### 4.4. TEM Imaging and Size Measurement

1 µL of fullerenol suspension was deposited onto a 3 mm copper grid with Formvar/Carbon support film (TedPella, Inc., Redding, CA, USA). The dried specimen was examined using a Hitachi HT7700 Exalens transmission electron microscope (Hitachi, Tokyo, Japan) operating in high-resolution (HR) mode at a 100 kV acceleration voltage and 15 μA current (Figure S1).

Fullerenol at 5 µg/mL in water and at 50 µg/mL in a complete culture medium was used and images were taken at a magnification of 30,000. Acquired images in TIFF format were analyzed using Fiji (ImageJ 1.54p) to measure particle diameter in nm. The microscope was calibrated to 1.974 nm/pixel at 10,000 magnification prior to imaging. The particle diameter was measured manually using ImageJ and the measurement results were exported and then analyzed.

### 4.5. IR Analysis

The IR spectrum of fullerenol was recorded using a Bruker Vertex 80V FTIR spectrometer (Bruker, Billerica, MA, USA). The sample was ground in an agate mortar and pressed into a pellet with KBr additive at a ratio of 1:300 for transmission measurements. The spectrum was acquired by averaging 16 scans at a resolution of 2 cm^−1^ over the wavelength range of 400–4000 cm^−1^.

### 4.6. Determination of Endotoxin Level

The quantitative content of endotoxin (lipopolysaccharide, a membrane component of Gram-negative bacteria) in the fullerenol solution was assessed using the LAL (Lyophilized Amebocyte Lysate) test (Chromogenic Endotoxin Quant Kit, Thermo Scientific, Waltham, MA, USA). This method is based on measuring the absorbance of the reaction mixture at 405 nm.

A calibration curve was constructed for the endotoxin range of 0.01 to 0.05 EU/mL. Fullerenol was dissolved in distilled water to a concentration of 10 μg/mL. The test was performed according to the manufacturer’s instructions with two replicates. All measured absorbance values were corrected for the absorbance of a negative control.

The endotoxin content of fullerenol was determined using the following equation:

(1) $$C_{\mathrm{endo}}\;=\;\frac{C_{measured}}{C_{ful}}$$

where

*C_endo_*—concentration of endotoxin in a dry fullerenol sample ($\frac{EU}{mL}$).

*C_measured_*—concentration of endotoxin measured in the assay ($\frac{EU}{mL}$).

*C_ful_*—concentration of fullerenol used in the assay ($\frac{\mu g}{mL}$).

### 4.7. Absorbance Spectrum Measurement

Fullerenol C_60_(OH)_24_ was dissolved in deionized water at a concentration of 20 µg/mL. The solution was placed in a quartz cuvette with an optical path length of 1 cm, and the absorption spectrum was measured using a Multiskan Sky 1530-800560C spectrophotometer (Thermo Scientific, USA) over a wavelength range of 200 nm to 1000 nm with a step size of 1 nm. The absorption spectrum of the water used to dissolve the nanoparticles was also measured and subtracted from the sample spectrum.

### 4.8. Measurement of Metal Impurities

The trace element content was determined using inductively coupled plasma mass spectrometry (ICP-MS) with a Bruker AURORA M90 spectrometer (USA). Element concentrations in the samples were calculated using the spectrometer firmware. The accuracy of the measurements was verified using the reference standard “Trace Metals in Drinking Water” from High-Purity Standards (Charleston, VA, USA). Laboratory analyses were carried out at the Shared Research Facilities Center of Perm State National Research University.

### 4.9. Fluorescence Spectrum

The fluorescence spectrum was measured using a Synergy H1 hybrid reader (BioTek Instruments, Winooski, VT, USA) at 37 °C. Measurements were carried out across an excitation wavelength (λ_exc_) range of 270 nm to 650 nm with 20 nm increments. Fluorescence intensity was recorded in the range of λ_exc_ + 30 nm up to 700 nm. For the measurement, 200 μL of an aqueous solution of fullerenol C_60_(OH)_24_ (200 μg/mL) was added to a well of a black 96-well polystyrene microplate.

### 4.10. Cell Culturing

THP-1 cells in CCM were seeded at a density of 50,000 cells per well in 96-well plates (SPL, Pocheon-si, Republic of Korea). Fullerenol was then added to achieve final concentrations of 0.25, 2.5, 25, 50, 100, 250, 500, 750, and 1000 µg/mL. The cells were incubated with fullerenol for 24, 48, and 72 h in a humidified incubator maintained at 37 °C with 5% CO_2_. Four independent experiments were conducted on different days (*n* = 4).

### 4.11. Flow Cytometry

At the end of the incubation period, cells were transferred to tubes for flow cytometry and stained with the live/dead dye Zombie Aqua (ZA) (Invitrogen, Carlsbad, CA, USA). Following washing with phosphate-buffered saline (PBS) containing 10% bovine serum albumin (BSA), cells were stained with Annexin V FITC (AnnV) (BioLegend, San Diego, CA, USA) according to the manufacturer’s protocol. After staining, samples were kept on ice. Flow cytometric analysis was performed immediately using a CytoFlex S flow cytometer (Beckman Coulter, Brea, CA, USA) to determine the percentages of ZA^−^AnnV^+^ (early apoptotic) and ZA^+^AnnV^+^ (late apoptotic/necrotic) cells. Data were analyzed with CytExpert 2.4 software (Beckman Coulter, USA).

**Figure 16:**
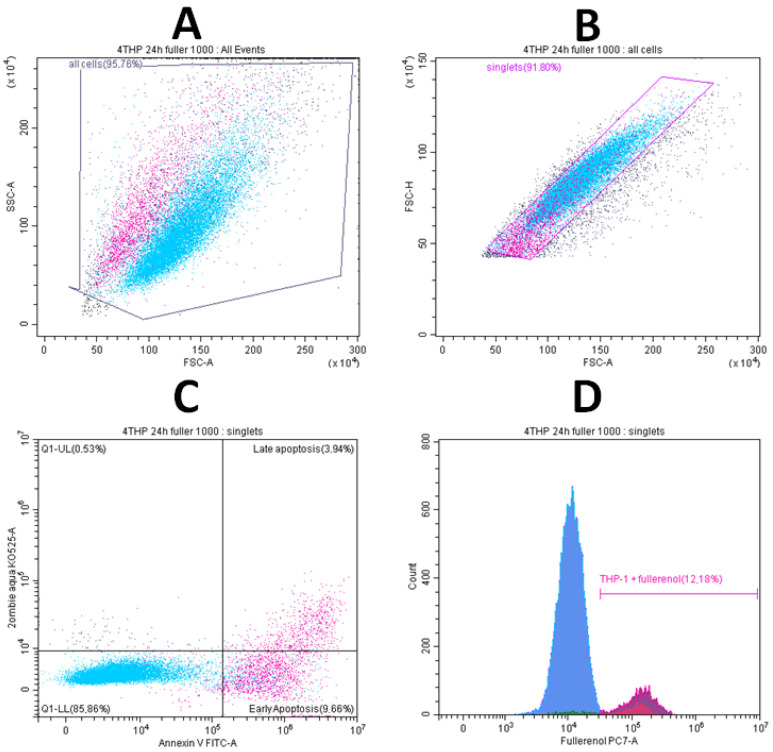
Gating strategy for determining the viability of THP-1 cell line cells and the adhesion/internalization of fullerenol by these cells. (**A**)—selection of the cell gate; (**B**)—gating of singlets; (**C**)—gating of cells in early (ZA^−^AnnV^+^) and late (ZA^+^AnnV^+^) apoptosis; (**D**)—detection of fullerenol association in the PC7 dye channel.

Adhesion and internalization of fullerenol nanoparticles were assessed by measuring the autofluorescence intensity of cells in the phycoerythrin-cyanine 7 (PE-Cy7, PC7) fluorescence channel (λ_ex_ = 488 nm; bandpass filter: 720–840 nm). Results are expressed as the median fluorescence intensity (MFI) in the PC7 channel of the target cell population (Figure 16).

### 4.12. Cell Metabolism

**Figure 17:**
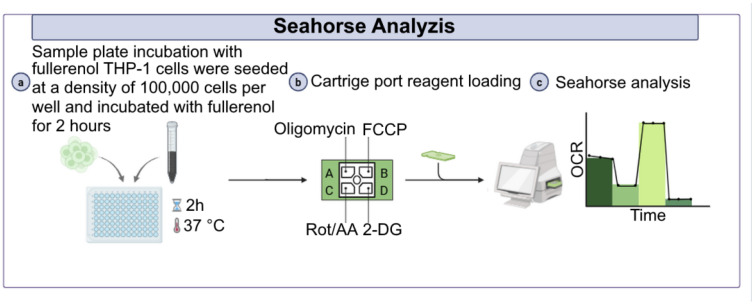
Design of the experiment to assess the metabolism of THP-1 cells under the influence of fullerenol C_60_(OH)_24_.

To assess the effects of fullerenol nanoparticles on THP-1 cell metabolism, cells were incubated with fullerenol for 24 h as described previously. On the day of the experiment we additionally measured viability of THP-1 cells using the trypan blue (BioinnLabs, Rostov-on-Don, Russia) dye exclusion method. After that the cells were resuspended in XF RPMI Medium (Agilent Technologies, USA) supplemented with 10 mM glucose (Sigma, Burlington, MA, USA), 2 mM glutamine (Capricorn, Germany), and 1 mM sodium pyruvate (Merck, Darmstadt, Germany). A total of 1 × 10^5^ cells were added to the wells of a 96-well plate (Agilent Technologies, USA), which had been pre-coated with poly-D-lysine, and centrifuged for 4 min at 200× *g* to form a monolayer. Subsequently, fullerenol nanoparticles were added to the wells to final particle concentrations of 2.5, 25, 50, 100, 200 and 750 μg/mL and incubated at 37 °C for 120 min (Figure 17).

Mitochondrial respiration and glycolysis were assessed by measuring the oxygen consumption rate (OCR) and extracellular acidification rate (ECAR), respectively, using Seahorse analyzer XFe96 (Agilent Technologies, USA). Measurements were taken at the basal level, followed by the sequential addition of mitochondrial respiratory chain inhibitors oligomycin 2.5 μM, carbonyl cyanide-4-(trifluoromethoxy) phenylhydrazone (FCCP; 2 μM), rotenone and antimycin A (R/A; 0.5 μM), and a competitive glycolysis inhibitor 2-dihydroxy-D-glucose (2 DG; 50 mM) (all from Agilent Technologies, USA). Data analysis was performed using Wave Desktop software version 2.6 (Agilent Technologies, USA).

To study the effect of fullerenol nanoparticles on the OXPHOS in THP-1 cells, the following parameters were determined: non-mitochondrial respiration (defined as the minimum OCR following R/A injection); basal OCR (calculated as the last OCR measurement before oligomycin injection minus non-mitochondrial respiration); max OCR (the first OCR measurement after FCCP injection minus non-mitochondrial respiration); and spare respiratory capacity (SRC), defined as the difference between max and basal OCR. Basal OCR represents mitochondrial respiration under resting conditions, maximal OCR reflects the highest oxygen consumption capacity of the cells, and SRC indicates the cells’ ability to increase respiration in response to elevated energy demand.

To evaluate glycolysis, several extracellular acidification rate (ECAR) parameters were measured: non-glycolytic acidification, defined as the minimum ECAR observed after 2-deoxyglucose (2-DG) injection, which reflects acidification from sources other than glycolysis; basal ECAR, calculated as the third ECAR measurement minus non-glycolytic acidification, representing glycolytic activity under baseline conditions; maximal ECAR, defined as the highest ECAR following rotenone and antimycin A (R/A) injection minus non-glycolytic acidification, indicating the peak glycolytic capacity; and compensatory glycolysis, calculated as the difference between maximal and basal ECAR, reflecting the cell’s ability to increase glycolysis when mitochondrial respiration is inhibited.

To improve the glycolytic rate estimation, all ECAR values were also transformed into proton efflux rate (PER), which takes into account the buffer capacity of the medium, according to the manufacturer’s instructions [43].

### 4.13. Statistical Analysis

Statistical analysis was performed using GraphPad Prism version 8.0.1. Data normality was assessed with the Shapiro–Wilk test. For normally distributed data, comparisons were made using the Welch/Brown–Forsythe test (nanoparticles association) or repeated measures ANOVA (metabolism). For non-parametric data, the Friedman test followed by Dunn’s post hoc test for multiple comparisons was applied. A *p*-value of less than 0.05 was considered statistically significant.

### 4.14. Limitations of the Method

The limitation of using flow cytometry for our studies is the impossibility of separating the processes of adhesion and internalization of fullerenol. In addition, at this stage we cannot say what size of fullerenol aggregates are formed in the medium during cultivation, and how aggregation can affect the fluorescence indices of cells with nanoparticles in the channel of the device we used.

## 5. Conclusions

Thus, it was established that the fullerenol nanoparticles C_60_(OH)_24_ used in this study exhibited physico-chemical properties typical for fullerenol. Our results demonstrate that they do not exhibit cytotoxic effects on THP-1 cells at concentrations up to 500 μg/mL. However, at higher concentrations, a decrease in cell viability and an increase in apoptosis—primarily early apoptosis—were observed, accompanied by a reduction in glycolytic activity. These findings confirm that the cytotoxicity of fullerenol depends on both concentration and incubation time, as well as the likely uptake of nanoparticles by the cells. Future studies should investigate additional parameters of THP-1 cells under fullerenol exposure, particularly metabolic activity, as well as the effects on other immune cell types. Based on the current data, the use of particle concentrations above 500 μg/mL is not recommended when assessing safe exposure levels of fullerenol for immune cells.

## Figures and Tables

**Table 1:** Correlation between median fluorescence intensity of fullerenol and cell parameters.

Analyzed Parameters	Time, h	Spearman’s Correlation Coefficient (95% Confidence Interval)	*p* Value
Fullerenol association and cell viability	24	0.04 (−0.29–0.35)	0.8137
	48	−0.43 (−0.66–(−0.13))	0.0057
	72	−0.78 (−0.88–(−0.61))	<0.0001
Fullerenol association and apoptosis	24	0.57 (0.31–0.76)	0.0001
	48	0.86 (0.75–0.93)	<0.0001
	72	0.88 (0.78–0.94)	<0.0001

**Table 2:** Comparative characterization of the effects of fullerenol C_60_(OH)_24_ on immune system cells.

Parameters	Time, h		
	24	48	72
**NK-cells (0.25, 0.5, 2.5, 5, 12.5, 25, 50, 100, 200 µg/mL) (CD3^−^CD56^+^)**			
Viability, % and absolute quantity	↔	↔	↔
Fullerenol association			↑100–200
**T-cells (0.25, 0.5, 2.5, 5, 12.5, 25, 50, 100, 200 µg/mL) (CD3^+^)**			
Viability, %	↓100–200	↓200	↔
Viability, absolute quantity	↔	↔	↓50–200
Fullerenol association	↑200	↑100–200	↑100–200
**B-cells (0.25, 0.5, 2.5, 5, 12.5, 25, 50, 100, 200 µg/mL) (CD19^+^)**			
Viability, % and absolute quantity	↔	↔	↔
Fullerenol association	↑200	↑100–200	↑50–200
**Monocytes (0.25, 0.5, 2.5, 5, 12.5, 25, 50, 100, 200 µg/mL) (CD14^+^)**			
Viability, % and absolute quantity	↓200	↓200	↔
Fullerenol association	↑12.5–200	↑2.5–200	↑25–200
**THP-1 (0.25, 2.5, 25, 50, 100, 250, 500, 750, 1000 µg/mL)**			
Viability, %	↑750	↔	↓750–1000
Early apoptosis, %	↔	↔	↑750–1000
Late apoptosis, %	↑1000	↑750–1000	↑750–1000
Fullerenol association	↑250–1000	↑250–1000	↑250–1000

↔—no effect; ↑—parameter increase; ↓—parameter decrease. The table presents statistically significant differences compared to the control without nanoparticles (*p* < 0.05). Detailed data are provided in publications [24,25,26].

## Data Availability

Datasets are available from the corresponding author on reasonable request.

## Supplementary Materials

The following supporting information can be downloaded at https://www.mdpi.com/article/10.3390/molecules30224407/s1, Figure S1: TEM images of fullerenol C_60_(OH)_24_.

## Abbreviations

The following abbreviations are used in this manuscript:

DLS	Dynamic light scattering
ECAR	Compensatory extracellular acidification rate
FCCP	Carbonyl cyanide-4-(trifluoromethoxy) phenylhydrazone
MFI	Mean fluorescence intensity
OCR	Oxygen consumption rates
PC7	Phycoerythrin-cyanine 7
PDI	Polydispersity index
PER	Proton efflux rate
SRC	Spare respiratory capacity
