# Supplementary material for: Effect of Fullerenol C60(OH)24 on the Viability and Metabolism of THP-1 Cells
Source: Molecules. 2025 Nov 14;30(22):4407. doi: 10.3390/molecules30224407 (PMC12655214; doi:10.3390/molecules30224407)
Supplement: Supplementary file 1 [file molecules-30-04407-s001.zip › molecules-3935660-supplementary.pdf]

## Figure S1: TEM images of fullerenol $C_{60}(OH)_{24}$

## Images used for size measurement

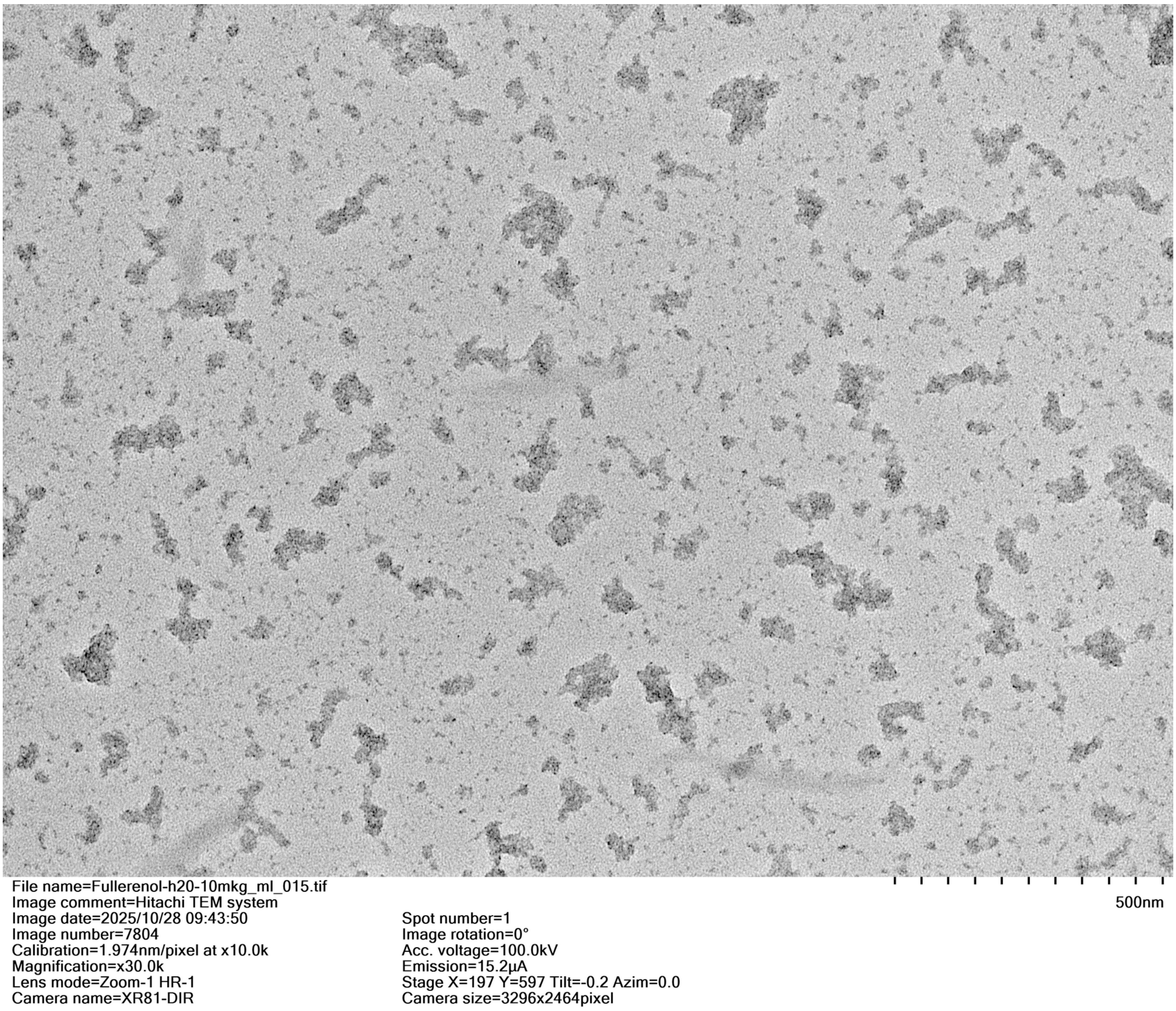


Fullerenol nanoparticles in water at 10 µg/mL

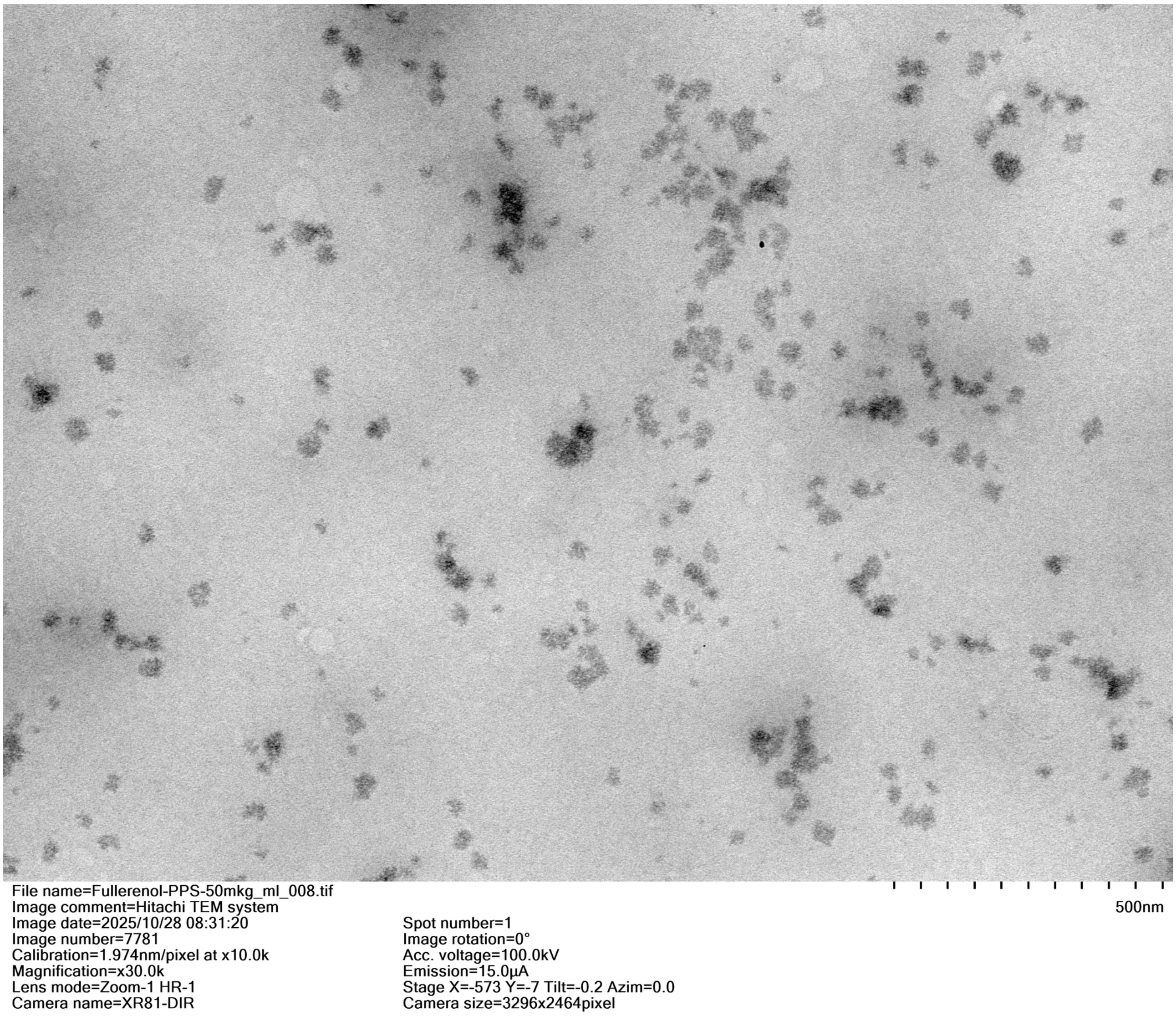


Fullerenol nanoparticles in complete culture medium at 50 µg/mL
